# Preclinical in vivo application of ^152^Tb-DOTANOC: a radiolanthanide for PET imaging

**DOI:** 10.1186/s13550-016-0189-4

**Published:** 2016-04-23

**Authors:** Cristina Müller, Christiaan Vermeulen, Karl Johnston, Ulli Köster, Raffaella Schmid, Andreas Türler, Nicholas P. van der Meulen

**Affiliations:** Center for Radiopharmaceutical Sciences ETH-PSI-USZ, Paul Scherrer Institut, Villigen-PSI, Switzerland; ISOLDE/CERN, Meyrin, Switzerland; Institut Laue-Langevin, Grenoble, France; Laboratory of Radiochemistry, Paul Scherrer Institut, Villigen-PSI, Switzerland; Department of Chemistry and Biochemistry, University of Bern, Bern, Switzerland

**Keywords:** ^152^Tb, PET, DOTANOC, Tumor imaging, AR42J tumors, ISOLDE

## Abstract

**Background:**

Terbium has attracted the attention of researchers and physicians due to the existence of four medically interesting radionuclides, potentially useful for SPECT and PET imaging, as well as for α- and β^−^-radionuclide therapy. The aim of this study was to produce ^152^Tb (*T*_1/2_ = 17.5 h, E_β+av_ = 1140 keV) and evaluate it in a preclinical setting in order to demonstrate its potential for PET imaging. For this purpose, DOTANOC was used for targeting the somatostatin receptor in AR42J tumor-bearing mice.

**Methods:**

^152^Tb was produced by proton-induced spallation of tantalum targets, followed by an online isotope separation process at ISOLDE/CERN. After separation of ^152^Tb using cation exchange chromatography, it was directly employed for radiolabeling of DOTANOC. PET/CT scans were performed with AR42J tumor-bearing mice at different time points after injection of ^152^Tb-DOTANOC which was applied at variable molar peptide amounts. ^177^Lu-DOTANOC was prepared and used in biodistribution and SPECT/CT imaging studies for comparison with the PET results.

**Results:**

After purification, ^152^Tb was obtained at activities up to ~600 MBq. Radiolabeling of DOTANOC was achieved at a specific activity of 10 MBq/nmol with a radiochemical purity >98 %. The PET/CT scans of mice allowed visualization of AR42J tumor xenografts and the kidneys, in which the radiopeptide was accumulated. After injection of large peptide amounts, the tumor uptake was reduced as compared to the result after injection of small peptide amounts. PET images of mice, which received ^152^Tb-DOTANOC at small peptide amounts, revealed the best tumor-to-kidney ratios. The data obtained with ^177^Lu-DOTANOC in biodistribution and SPECT/CT imaging studies confirmed the ^152^Tb-based PET results.

**Conclusions:**

Production of 30-fold higher quantities of ^152^Tb as compared to the previously performed pilot study was feasible. This allowed, for the first time, labeling of a peptide at a reasonable specific activity and subsequent application for in vivo PET imaging. As a β^+^-particle-emitting radiolanthanide, ^152^Tb would be of distinct value for clinical application, as it may allow exact prediction of the tissue distribution of therapeutic radiolanthanides.

**Electronic supplementary material:**

The online version of this article (doi:10.1186/s13550-016-0189-4) contains supplementary material, which is available to authorized users.

## Background

Terbium has attracted the attention of researchers in radiopharmaceutical sciences due to the existence of four medically interesting radionuclides for SPECT (^155^Tb) and PET (^152^Tb) imaging, as well as for α- (^149^Tb) and β^−^-radionuclide (^161^Tb) therapy. The first proof-of-concept study, using all four Tb radionuclides in combination with a folate-based tumor targeting agent, has been reported by Müller et al. [1]. It was demonstrated that the production and preclinical application of these four Tb nuclides is feasible. Of the four Tb nuclides, ^161^Tb is the most advanced with regard to the production and separation methods, previously introduced by Zhernosekov et al. [2] and currently implemented at PSI [1]. In vivo studies have been performed in order to assess the tissue distribution profile of ^161^Tb-labeled biomolecules and to investigate the therapeutic potential of ^161^Tb in comparison to the clinically- established ^177^Lu [3–5]. Based on the results of these studies, it is likely that the co-emitted Auger and conversion electrons of ^161^Tb contributed positively to the overall anti-tumor effects, while additional side effects to the kidneys were not observed [6]. Another interesting Tb nuclide for therapeutic purposes is ^149^Tb, which decays with a half-life of 4 h and the emission of α-particles. Due to the currently limited availability of this radionuclide, extended in vitro and in vivo studies have not been performed yet. There is, however, a clear indication of its potential for α-radionuclide therapy, as demonstrated in preclinical proof-of-concept studies with ^149^Tb-labeled rituximab [7] as well as with a ^149^Tb-labeled DOTA-folate conjugate [8]. Just recently, we demonstrated that ^149^Tb can also be used for PET imaging based on the emission of β^+^-particles [9].

Two other Tb nuclides, ^155^Tb and ^152^Tb, are suited for diagnostic applications. Recently, preclinical experiments were performed in order to investigate ^155^Tb in more detail with regard to its potential for SPECT imaging [10]. For this purpose, several DOTA-functionalized tumor targeting agents, among those also peptides and an antibody, were employed for imaging of tumors in mice [10]. The image quality of the ^155^Tb-based SPECT scans was convincing and allowed not only the visualization of subcutaneous tumor xenografts, but also smallest lesions in the abdominal tract of a mouse with intraperitoneal SKOV-3ip xenografts [10]. ^155^Tb-based SPECT imaging was also investigated using a Derenzo phantom, which proved that the achievable image resolution was comparable to the resolution of ^111^In, a clinically well-established SPECT nuclide [10].

Since PET is commonly the preferred imaging technique in nuclear medicine, the development of new PET nuclides is of general interest. In this regard, ^152^Tb is a promising candidate to be used for PET imaging based on the emission of β^+^-particles, while co-emission of therapeutic particles is absent (Table 1). The relatively long half-life of 17.5 h appears particularly attractive, as it may allow pre-therapeutic imaging, potentially useful for dosimetry calculations prior to therapy with particle-emitting radiolanthanides, such as ^177^Lu, ^166^Ho, ^161^Tb, and ^149^Tb. The limited availability of ^152^Tb has, however, prevented further in vivo imaging studies to date.

**Table 1:** Decay properties of ^152^Tb based on NuDat 2.6 (www.nndc.bnl.gov/nudat2/)

Nuclide	Half-life	Eβ^+^_ average_ (I)	Significant γ-rays (I > 5 %); Eγ (I)	Application
^152^Tb	17.5 h	1140 (20.3 %)	271 (9.53 %); 344 (63.5 %); 586 (9.21 %); 779 (5.54 %)	PET imaging

**Fig. 1:**
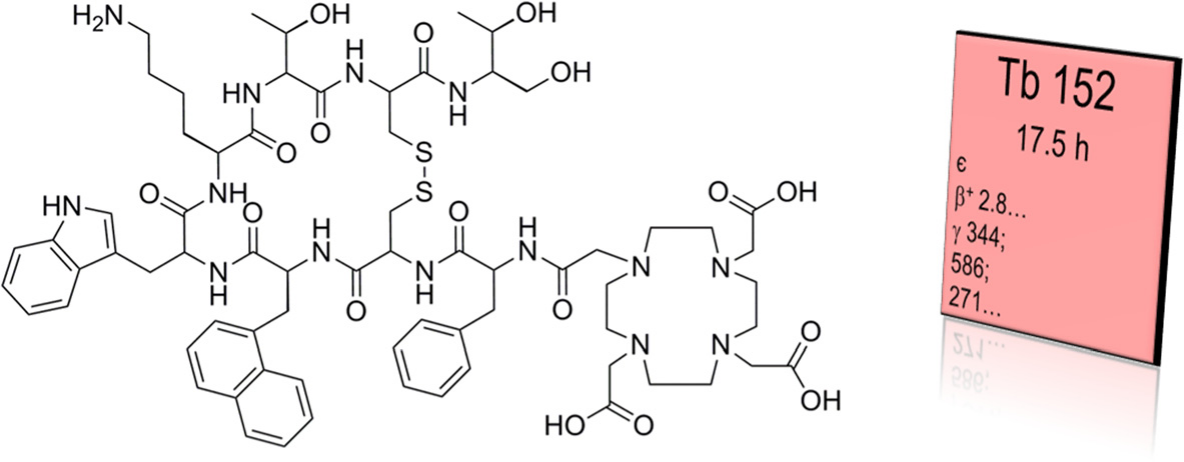
Chemical structure of DOTANOC which was radiolabeled with ^152^Tb for PET, along with the radiation properties of ^152^Tb as obtained from the Karlsruhe Nuclide Chart (www.nucleonica.com)

The aim of this study was to produce ^152^Tb at larger quantities at the ISOL facility at CERN/ISOLDE and process it to allow using it for in vivo studies. For this purpose, DOTANOC, a clinically relevant peptide for targeting the somatostatin receptor, was employed (Fig. 1) [11–13].

Tumor-bearing mice were injected with the ^152^Tb-labeled peptide in order to assess the potential of ^152^Tb for PET imaging using a preclinical PET/CT scanner. The data obtained with ^152^Tb-DOTANOC were compared with the tissue distribution data and SPECT image obtained with the ^177^Lu-labeled counterpart in order to corroborate the hypothesis that ^152^Tb could serve as a surrogate for therapeutic radiolanthanides.

## Methods

### Production of ^152^Tb

^152^Tb was produced by 1.4 GeV proton-induced spallation in a tantalum foil target (55 g/cm^2^) at ISOLDE (CERN, Geneva, Switzerland). The spallation products were released online from the hot (~2000 °C) target and ionized in a hot (~2000 °C) tungsten ionizer. The cumulative ^152^Tb yields were significantly boosted by resonant laser ionization [14] of the radioactive precursor ^152^Dy. The ions were then accelerated to 30 keV and mass-separated, as previously reported [1, 15]. Mass 152 ions were implanted into zinc-coated gold foils.

### Separation of ^152^Tb from target material and impurities

The zinc layer containing ^152^Tb, and other mass 152 impurities, was dissolved in 0.1 M HNO_3_/NH_4_NO_3_ at 80 °C. After addition of H_2_O, the solution was passed through a 50 mm × 5 mm column containing a macroporous strongly acidic cation exchange resin, as previously described [1]. A concentration gradient of α-hydroxyisobutyric acid (α-HIBA; pH 4.7) ranging from 0.07, 0.09, 0.11 to 0.13 M was used for elution. The gradient pump was set to run at 0.33 mL/min, and ~1 mL fractions were collected in pre-washed Eppendorf tubes. Fractions containing the highest activity of ^152^Tb were used directly in the subsequent radiolabeling of DOTANOC.

### Radiolabeling of DOTANOC

DOTANOC was obtained from ABX GmbH (DOTA-NOC acetate Cat-N° 9712). The peptide was dissolved in MilliQ water to obtain a stock solution of 1 mM. The radiolabeling with ^152^Tb was performed by direct addition of 12 μL DOTANOC stock solution to a ^152^Tb-α-HIBA solution (120 MBq in ~500 μL, pH 4.7). The reaction mixture was incubated at 95 °C for 15 min. Labeling of DOTANOC with n.c.a. ^177^Lu (Isotope Technologies Garching ITG GmbH, Germany) was performed under standard conditions in a mixture of HCl (0.05 M) and Na-acetate (0.5 M) at pH 4.5 and elevated temperature (15 min, 95 °C). High-performance liquid chromatography (HPLC) with a C-18 reversed-phase column (Xterra™ MS, C18, 5 μm, 150 × 4.6 mm; Waters) was used for quality control of the radiolabeled peptides. The mobile phase consisted of MilliQ water containing 0.1 % trifluoroacetic acid (A) and acetonitrile (B). A gradient from 95 % A and 5 % B to 20 % A and 80 % B over a period of 15 min was used with a flow rate of 1.0 mL/min. The product peak corresponding to ^152^Tb-DOTANOC and ^177^Lu-DOTANOC, respectively, appeared with a retention time (*R*_t_) of 11.1 min.

### Cell culture

AR42J tumor cells, a rat exocrine pancreatic tumor cell line which is known to express the somatostatin receptor, were obtained from the European Collection of Cell Cultures (ECACC). The cells were cultured in RPMI 1640 medium, supplemented with 10 % fetal calf serum, l-glutamine, and antibiotics under standard conditions in a humidified atmosphere with 5 % CO_2_ at 37 °C. Routine culture was performed by trypsinization of the cells twice a week. To inoculate the animals, the cells were detached using trypsin, centrifuged, and resuspended in PBS at a concentration of 5 × 10^6^ cells/100 μL.

### Tumor mouse model

In vivo experiments were approved by the local veterinary department and conducted in accordance with the Swiss law of animal protection. Female nude mice (CD-1 Foxn-1/nu) were purchased from Charles River Laboratories (Sulzfeld, Germany). The animals were fed with a standard rodent chow ad libitum (Kliba Nafag, Kaiseraugst, Switzerland).

About 2 weeks prior to biodistribution and imaging studies, the mice were inoculated with AR42J tumor cells by subcutaneous injection of the cell suspension (5 × 10^6^ cells/100 μL) on each shoulder.

### Biodistribution study of ^177^Lu-DOTANOC in AR42J tumor-bearing mice

Biodistribution studies were performed in triplicate in AR42J tumor-bearing nude mice. ^177^Lu-DOTANOC (5 MBq, 2.5 nmol per mouse) was administered intravenously (i.v.) into a tail vein. Mice were euthanized at pre-determined time points between 30 min and 22 h after injection of the radioconjugate. Two additional groups of three mice each were injected with 5 MBq ^177^Lu-DOTANOC and a peptide amount of 0.5 and 5.0 nmol, respectively. Selected tissues and organs were collected, weighed, and counted for radioactivity using a γ-counter (Packard Canberra Cobra II, USA). The results were listed as a percentage of the injected activity per gram of tissue mass (% IA/g), using counts of a defined volume of the original injection solution measured at the same time.

### SPECT/CT imaging studies

SPECT scans were performed after the injection of ^177^Lu-DOTANOC using a NanoSPECT/CT™ (Mediso Medical Imaging Systems, Budapest, Hungary) and Nucline Software (version 1.02, Bioscan Inc., Poway, USA). For this purpose, the energy peaks of ^177^Lu were set to 56.1 keV ± 10 %, 112.9 keV ± 10 %, and 208.4 keV ± 10 %. The acquired data were reconstructed using HiSPECT software (version 1.4.3049, Scivis GmbH, Göttingen, Germany). Images were prepared using the VivoQuant post-processing software (version 2.1, inviCRO Imaging Services and Software, Boston, USA). Accumulation of ^177^Lu-DOTANOC per volume of tumor and kidney tissue was determined using the “3D ROI” tool of the the *VivoQuant* post-processing software, allowing calculation of the tumor-to-kidney ratios. A Gauss post-reconstruction filter was applied for the presentation of the SPECT image, and the scale was adjusted to allow the best visualization of tumors and kidneys in which radioactivity accumulated.

### PET/CT imaging studies

PET scans were performed with a bench-top preclinical PET/CT scanner (G8, Sofie Biosciences, California, USA and Perkin Elmer, Massachusetts, USA). The energy window ranged from 150 to 650 keV and the spatial resolution of reconstructed images was 1.4 mm full-width-at-half-maximum in the center of the field of view [16]. The images were acquired as static whole-body scans using G8 acquisition software (version 1.2.9.3) and reconstructed with maximum-likelihood expectation maximization (MLEM). The data were corrected for random coincidences, decay and dead time. Images were prepared using the VivoQuant post-processing software (version 2.1, inviCRO Imaging Services and Software, Boston, USA). Accumulation of ^152^Tb-DOTANOC per volume of tumor and kidney tissue was determined using the “3D ROI” tool of the *VivoQuant* post-processing software, allowing calculation of the tumor-to-kidney ratios. A Gauss post-reconstruction filter was applied for the presentation of the PET, with the scale adjusted to allow the best visualization of tumors and kidneys in which radioactivity accumulated.

## Results

### Production of ^152^Tb

Samples of ~1.3 GBq ^152^Tb were collected after online mass separation at the ISOLDE facility of CERN and implanted into Zn plated on gold foils. Collections were performed for 4-h periods utilizing a 2 μA, 1.4 GeV proton beam on the spallation target. After leaving the collected samples to stand, to allow the short-lived precursor, ^152^Dy, as well as ^136^Nd and ^136^Pr which are present as oxide sidebands to decay, the samples were transported to PSI. Radiochemical separation was performed 18 h after end of collection, when ~650 MBq ^152^Tb remained.

### Separation of ^152^Tb

The radiochemical separation, based on cation exchange chromatography, saw the collection of 12 1-mL samples, yielding a total of ~600 MBq (>90 %) ^152^Tb in <2.5 mL 0.13 M α-HIBA. The gradient elution of the column at low pump speeds ensured the sole elution of ^152^Tb, leaving the remaining contaminants, predominantly Ce, to be eluted with more concentrated solutions. The ^152^Tb eluent was radiochemically pure and used directly for radiolabeling purposes.

### Preparation of radiopeptides

**Fig. 2:**
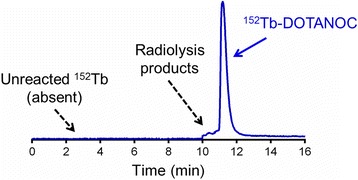
HPLC chromatogram of ^152^Tb-DOTANOC (*R*_t_ = 11.1 min, >98 % radiochemical purity) showing the absence of unreacted ^152^Tb(III) (*R*_t_ = 2.3 min, <1 %). Formation of radiolysis products are shown as a shoulder of the main peak (*R*_t_ = 10–11 min)

Radiolabeling of DOTANOC with ^152^Tb was achieved at a specific activity of 10 MBq/nmol and a radiochemical purity of >98 % as determined by radio-HPLC (Fig. 2). The chromatogram indicated the onset of radiolysis, evidenced by a shoulder (*R*_t_ = 10–11 min) before the product peak (*R*_t_ = 11.1 min). This peak area was not taken into consideration for determination of the radiochemical purity. Preparation of ^177^Lu-DOTANOC at a specific activity of 10 MBq/nmol was readily achieved with a radiochemical purity of >98 %.

The reaction mixture of ^152^Tb-DOTANOC and ^177^Lu-DOTANOC, respectively, was diluted with saline for in vivo application. Na-DTPA (5–10 μL, 5 mM, pH 5) was added for complexation of potential traces of unreacted ^152^Tb and ^177^Lu, respectively.

### Biodistribution study of ^177^Lu-DOTANOC in AR42J tumor-bearing mice

Biodistribution studies were performed in AR42J tumor-bearing nude mice at 30 min, 2 h, 5 h, 16 h, and 22 h after injection of ^177^Lu-DOTANOC (5 MBq/2.5 nmol) in order to quantify the tissue distribution of radioactivity (Table 3). Based on the data obtained in these experiments, the radioconjugate was quickly cleared from the blood and accumulated in the tumor tissue, where it reached a maximum uptake of 9.02 ± 0.33 % IA/g already 30 min after injection. Over the first 5 h, the uptake in the tumor remained almost constant, but it decreased later to 5.48 ± 0.98 % IA/g at 16 h and 4.51 ± 0.37 % IA/g at 22 h after injection. In the kidneys, the highest value of accumulated radioactivity (13.0 ± 1.85 % IA/g) was determined 30 min after injection of ^177^Lu-DOTANOC. The radioactivity was cleared from the kidneys over time, resulting in 4.80 ± 0.19 % IA/g at 22 h after injection. As a consequence, the tumor-to-kidney ratios were ~1 within the time window of 2 to 22 h after injection of ^177^Lu-DOTANOC applied at the mentioned molar peptide amount.

**Fig. 3:**
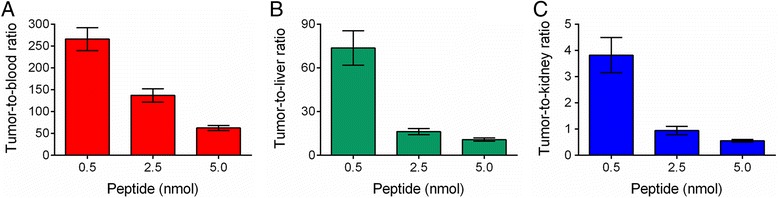
Tumor-to-background ratios of ^177^Lu-DOTANOC obtained after injection of three different molar amounts of injected peptide (0.5, 2.5, and 5.0 nmol). **a** Tumor-to-blood ratios, **b** tumor-to-liver ratios, and **c** tumor-to-kidney ratios

Additional experiments were conducted to investigate the influence of the injected peptide amount (0.5, 2.5, and 5.0 nmol, respectively) on the tissue distribution of the radiopeptide. For this purpose, mice were euthanized and dissected 5 h after injection of ^177^Lu-DOTANOC. It was found that the absolute tumor uptake was highest (25.3 ± 4.14 % IA/g) in mice which received the lowest quantity of injected peptide (0.5 nmol), over threefold reduced (8.45 ± 1.04 % IA/g) in mice which received 2.5 nmol peptide and almost sevenfold reduced (3.82 ± 0.20 % IA/g) in mice injected with 5.0 nmol peptide. Accumulation of radioactivity in the kidneys was in the range of ~6.6 % IA/g to ~9.1 % IA/g, 5 h after injection of ^177^Lu-DOTANOC, independent of the molar amount of injected peptide. It was, thus, revealed that the tumor-to-kidney ratios were more favorable with the injection of small molar amounts of peptide than after injection of large quantities. The same holds true for tumor-to-blood and tumor-to-liver ratios (Fig. 3).

### PET/CT and SPECT/CT imaging studies

**Fig. 4:**
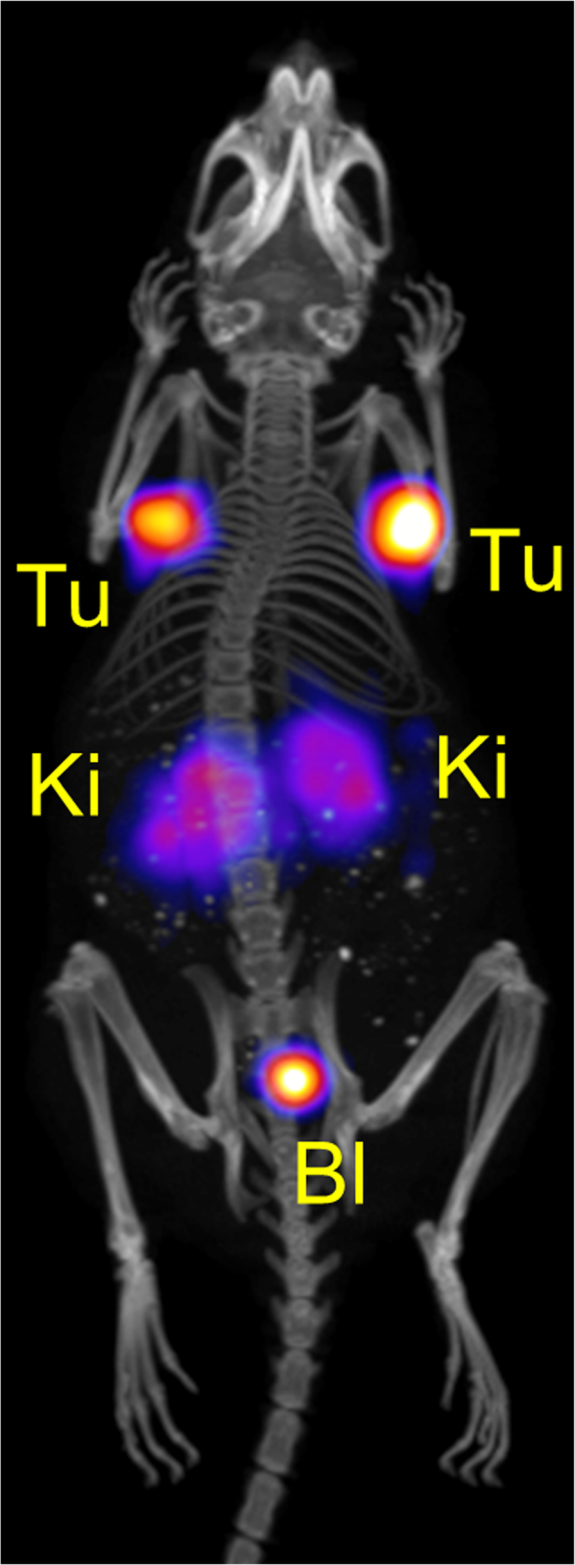
PET/CT image shown as maximal intensity projection of an AR42J tumor-bearing mouse 2 h after injection of Tb-DOTANOC (3.4 MBq; 0.34 nmol DOTANOC). During the in vivo scan the mouse was anesthetized with a mixture of isoflurane and oxygen. The image is presented with the scale adapted to allow visualization of tumors and kidneys. (*Tu* = AR42J tumor xenograft, *Ki* = kidney, *Bl* = urinary bladder)

In vivo PET/CT studies were performed with a preclinical G8 benchtop PET/CT scanner, which allowed acquisition of high-quality PET/CT images at low activities. Imaging studies were performed with AR42J tumor-bearing mice at variable time points after injection of different quantities of activity and molar amounts of ^152^Tb-DOTANOC (Table 2). In order to investigate the tissue distribution at an early time point, 3.4 MBq ^152^Tb-DOTANOC were injected, corresponding to 0.34 nmol DOTANOC. The mouse (M1, Table 2) was scanned 2 h after injection of ^152^Tb-DOTANOC. At this time, the activity in the animal was only ~0.52 MBq, which is an optimum quantity to perform PET scans with the highly sensitive G8 scanner. The in vivo PET scan of the mouse lasted for 20 min and was followed by a CT scan of 1.5 min. The resulting PET/CT image showed high uptake of radioactivity in AR42J tumor xenografts and, as a consequence of the excretion of ^152^Tb-DOTANOC, retention of activity was also found in the kidneys as well as in the urinary bladder (Fig. 4). The tumor-to-kidney ratio determined using VivoQuant software revealed a value of ~1.5. The increased tumor-to-kidney ratio after injection of low molar amounts of DOTANOC was in agreement with the results of the biodistribution study using ^177^Lu-DOTANOC, where the tumor-to-kidney ratio was found to be ~3.8 at 5 h after injection of low molar peptide amounts (0.5 nmol) (Fig. 3, Additional file 1: Table S1).

**Table 2:** Experimental design of the PET/CT and SPECT/CT imaging studies

Mouse	Radiopeptide	Injected activity (IA)	Injected DOTANOC	Scan start (scan duration)	Radioactivity at scan start	Imaging method	Figure
M1	^152^Tb-DOTANOC	~3.4 MBq	0.34 nmol	2 h p.i. (20 min)	~0.52 MBq	PET/CT	Fig. 4
M2	^152^Tb-DOTANOC	~25 MBq	2.5 nmol	5 h p.i. (20 min)	~1.87 MBq	PET/CT	Fig. 5a
				8 h p.i. (20 min)	~1.46 MBq	PET/CT	Fig. 5b
				22 h p.i. (20 min)	~0.44 MBq	PET/CT	Fig. 5c
M3	^152^Tb-DOTANOC	~47 MBq	4.7 nmol	22 h p.i. (30 min)	~0.83 MBq	PET/CT	Fig. 6a
				32 h p.i. (30 min)	~0.45 MBq	PET/CT	Additional file 1: Fig. S1^a^
M4	^177^Lu-DOTANOC	~47 MBq	4.7 nmol	22 h p.i. (4 h)^b^	~2.5 MBq	SPECT/CT	Fig. 6b

^a^

^b^Post-mortem scan

**Fig. 5:**
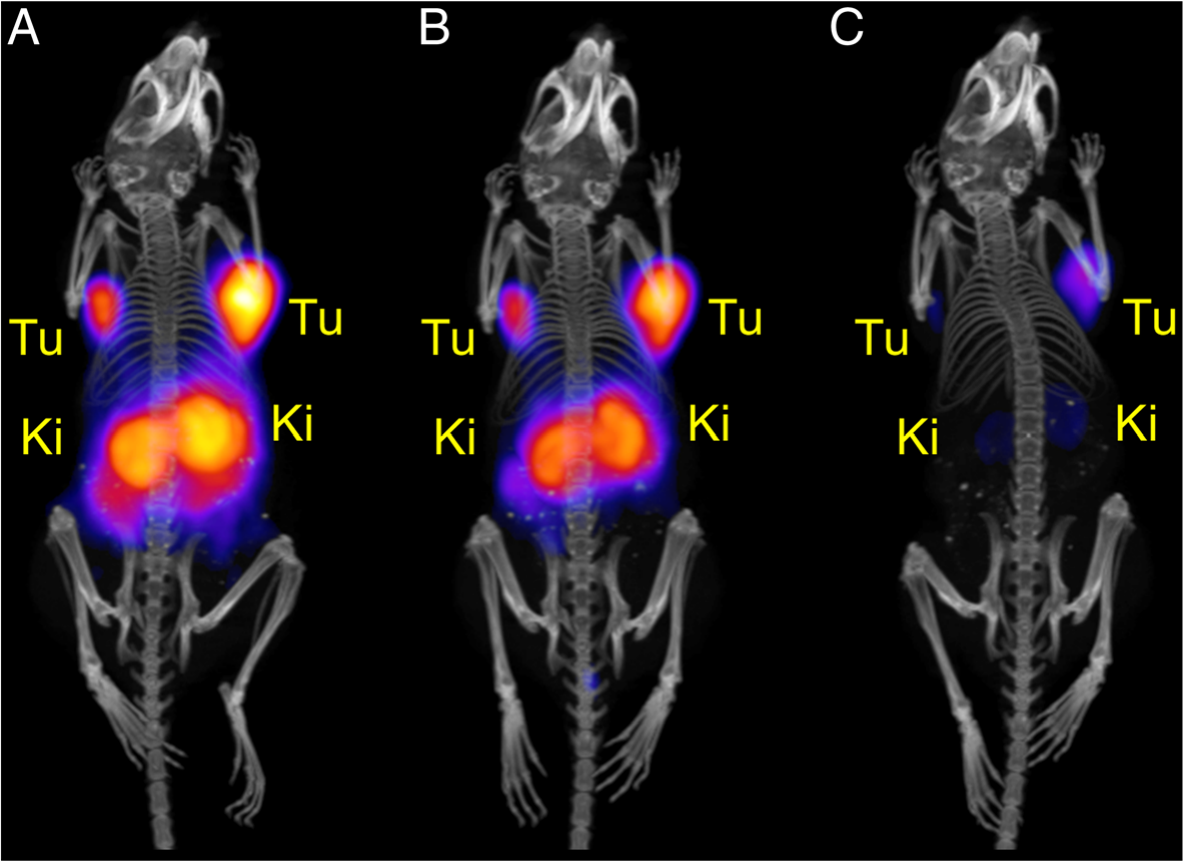
**a**–**c** PET/CT images shown as maximal intensity projections of an AR42J tumor-bearing mouse at different time points after injection of ^152^Tb-DOTANOC (25 MBq; 2.5 nmol DOTANOC). **a** PET/CT image at 5 h after injection of ^152^Tb-DOTANOC; **b** PET/CT image at 8 h after injection of ^152^Tb-DOTANOC, and **c** PET/CT image at 22 h after injection of ^152^Tb-DOTANOC. During the in vivo scan, the mouse was anesthetized with a mixture of isoflurane and oxygen. The images are presented with the scale kept at an equal level for all time points in order to reflect the effective activity retained in the mouse. (*Tu* AR42J tumor xenograft, *Ki* kidney)

In a subsequent experiment, the tissue distribution was investigated at later time points after injection. Another mouse (M2, Table 2) was, therefore, injected with a larger quantity of ^152^Tb-DOTANOC (25 MBq), which corresponded to a molar amount of 2.5 nmol peptide. The same molar amount was also used in biodistribution studies with ^177^Lu-DOTANOC (Table 3). The in vivo PET scans, which were performed at 5, 8, and 22 h after injection, lasted for 20 min and were followed by a CT scan of 1.5 min. At these time points, the activity in the mouse was reduced to ~1.87, ~1.46, and ~0.44 MBq, respectively. The PET/CT images of the mouse showed significant accumulation of radioactivity in AR42J tumor xenografts located on each shoulder, as well as in the kidneys (Fig. 5a–c). Determination of the tumor-to-kidney ratios using VivoQuant software revealed values in the range of ~1 at all investigated time points (~1.1 at 5 h p.i., ~1.1 at 8 h p.i., and ~1.2 at 22 h p.i.). The findings were in good agreement with the values (~0.94) obtained in biodistribution studies performed in the same tumor mouse model after injection of the same molar amount of ^177^Lu-DOTANOC (Table 3).

**Table 3:** Biodistribution of ^177^Lu-DOTANOC (5 MBq/2.5 nmol) in AR42J tumor-bearing female nude mice, expressed in percentage of total injected activity per gram tissue (% IA/g)

	^177^Lu-DOTANOC				
	30 min p.i.	2 h p.i.	5 h p.i.	16 h p.i.	22 h p.i.
Blood	2.52 ± 0.65	0.09 ± 0.01	0.06 ± 0.01	0.07 ± 0.01	0.03 ± 0.00
Heart	1.34 ± 0.35	0.08 ± 0.01	0.07 ± 0.01	0.07 ± 0.01	0.03 ± 0.00
Lung	3.04 ± 0.47	0.45 ± 0.03	0.41 ± 0.04	0.27 ± 0.05	0.21 ± 0.03
Spleen	0.77 ± 0.03	0.15 ± 0.01	0.18 ± 0.02	0.13 ± 0.04	0.09 ± 0.01
Kidneys	13.0 ± 1.85	7.67 ± 0.51	9.10 ± 1.66	6.13 ± 1.63	4.80 ± 0.19
Adrenals	1.38 ± 0.32	0.28 ± 0.14	0.37 ± 0.05	0.51 ± 0.40	0.42 ± 0.18
Stomach	1.95 ± 0.23	1.14 ± 0.04	1.21 ± 0.55	0.68 ± 0.09	0.60 ± 0.22
Pancreas	1.99 ± 0.07	1.09 ± 0.17	0.88 ± 0.11	0.34 ± 0.05	0.25 ± 0.03
Intestines	0.94 ± 0.12	0.25 ± 0.04	0.25 ± 0.03	0.16 ± 0.01	0.13 ± 0.03
Liver	1.43 ± 0.17	0.52 ± 0.04	0.53 ± 0.10	0.27 ± 0.05	0.20 ± 0.02
Muscle	0.48 ± 0.16	0.02 ± 0.00	0.02 ± 0.01	0.03 ± 0.00	0.01 ± 0.00
Bone	1.13 ± 0.26	0.17 ± 0.01	0.12 ± 0.02	0.12 ± 0.05	0.08 ± 0.01
Brain	0.06 ± 0.01	0.01 ± 0.00	0.02 ± 0.00	0.02 ± 0.01	0.01 ± 0.00
AR42J tumor	9.02 ± 0.33	8.32 ± 0.47	8.45 ± 1.04	5.48 ± 0.98	4.51 ± 0.37
Tumor-to-blood	3.76 ± 1.05	96.6 ± 8.79	137 ± 15.3	80.0 ± 8.30	180 ± 17.5
Tumor-to-liver	6.39 ± 0.89	16.1 ± 1.34	16.2 ± 2.10	21.0 ± 2.68	23.0 ± 2.96
Tumor-to-kidney	0.71 ± 0.11	1.09 ± 0.06	0.94 ± 0.16	0.93 ± 0.19	0.94 ± 0.02

Values shown represent the mean ± S.D. of data from three animals (*n* = 3) per cohort

**Fig. 6:**
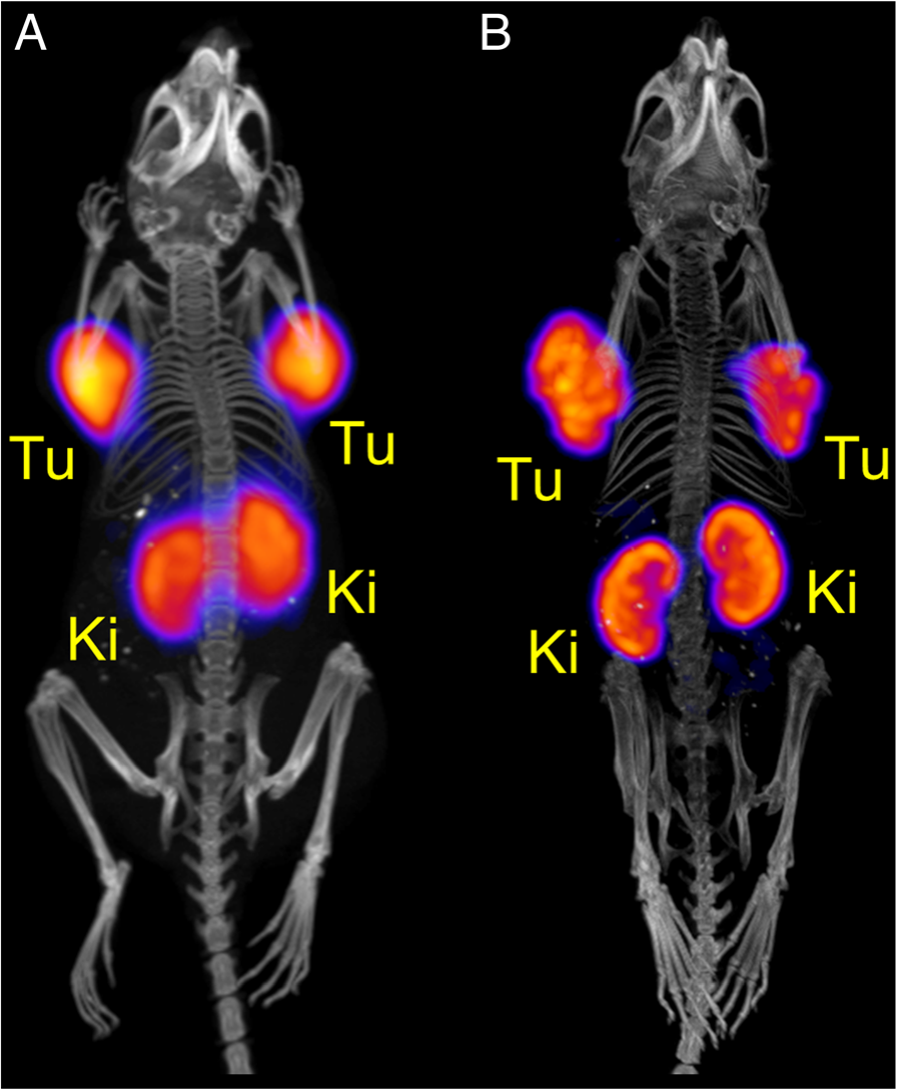
**a** PET/CT image and **b** SPECT/CT image shown as maximal intensity projections of AR42J tumor-bearing mice 22 h after injection of **a** ^152^Tb-DOTANOC (47 MBq; 4.7 nmol DOTANOC) and **b** ^177^Lu-DOTANOC (47 MBq; 4.7 nmol DOTANOC), respectively. The in vivo PET scan lasted for 20 min and was followed by a CT scan of 1.5 min. During the in vivo scan, the mouse was anesthetized with a mixture of isoflurane and oxygen. The post-mortem SPECT scan lasted for 4 h preceded by a CT scan of 7.5 min. (*Tu* AR42J tumor xenograft, *Ki* kidney)

A third mouse (M3, Table 2) was injected with even larger quantities of ^152^Tb-DOTANOC (47 MBq, 4.7 nmol DOTANOC) which would be ideal to perform scans at later time points after injection. The PET/CT scan was performed 22 h after injection of ^152^Tb-DOTANOC, when ~0.83 MBq were left in the animal. The PET scan lasted for 20 min and was followed by a CT scan of 1.5 min. To directly compare this with the tissue distribution of ^177^Lu-DOTANOC, a SPECT/CT scan was performed with a mouse which received ^177^Lu-DOTANOC at the same molar amount of peptide. At scan start, 22 h after injection, only ~2.5 MBq ^177^Lu were counted in this mouse. As this activity was too low for a short SPECT scan, the mouse was scanned for 4 h post-mortem in order to allow acquisition of sufficient counts. The CT, which was performed before the start of the SPECT scan, lasted for 7.5 min. The PET/CT and SPECT/CT images showed equal tissue distribution profiles of the two radioconjugates (Fig. 6a, b). Determination of the tumor-to-kidney ratios using the VivoQuant software revealed a value of ~1.0 for ^177^Lu-DOTANOC and ~1.1 was determined for ^152^Tb-DOTANOC. In biodistribution studies performed with ^177^Lu-DOTANOC, the relatively high molar amount of 5 nmol injected peptide resulted in a decreased tumor-to-kidney ratio (~0.55) at 5 h after injection of ^177^Lu-DOTANOC (Fig. 3, Additional file 1: Table S1). This indicates that, over time, the radioactivity was cleared faster from the kidneys than from tumors, resulting in increasing tumor-to-kidney ratios of ~1 at later time points after injection.

## Discussion

There are only few instances in preclinical studies where ^152^Tb has been used [1, 17]. The present study is the first in which ^152^Tb (available at ~30-fold higher quantities) could be employed for detailed in vivo PET imaging using tumor-bearing mice and a well-established somatostatin receptor targeting peptide. A unique and indisputable advantage of ^152^Tb, as compared to currently used ^68^Ga, is the possibility to use it for imaging purposes over a longer period. It may find application as a diagnostic and dosimetry match for clinically-used therapeutic radionuclides, such as ^177^Lu and ^166^Ho, or for the exact therapeutic matches ^161^Tb and ^149^Tb. In this regard, it may be of particular value in combination with targeting agents of a longer biological half-life such as albumin-binding small molecules or antibodies. Although other longer-lived PET nuclides such as ^89^Zr (*T*_1/2_ = 3.26 days) and ^64^Cu (*T*_1/2_ = 12.7 h) exist, these nuclides have different chemical properties and, hence, different chelators are necessary for stable coordination [18–20]. ^68^Ga is currently successfully used for PET imaging prior to ^177^Lu-based radionuclide therapy [11, 21], however, its short half-life and different coordination chemistry, which may result in different pharmacokinetics [22], would clearly speak in the favor of using a matched radiolanthanide for PET and, thus, justify the development and investigation of ^152^Tb.

In the proof-of-concept study in which ^152^Tb was used for the first time in vivo, only 20 MBq of ^152^Tb were available after separation, allowing labeling of a DOTA-folate conjugate at a low specific activity of 1.3 MBq/nmol [1]. As a consequence, relatively high molar amounts of the folate conjugate (~7 nmol) had to be injected in order to only obtain PET images at short time points (1.5 and 3 h) after injection, while imaging the following day was only possible post-mortem due to the long acquisition time which was required [1]. In the present study, a ~30-fold increased activity was obtained allowing radiolabeling at a markedly higher specific activity of 10 MBq/nmol. Such improvement was feasible due to significantly higher activities which were produced, allowing the performance of the radiolabeling with higher activity concentrations.

Partial degradation of the peptide, as a consequence of radiolysis, was observed as a shoulder of the product peak—a phenomenon which is more pronounced with increasing radioactivity concentrations. The addition of ascorbic acid or gentisic acid to the labeling solution would, most likely, prevent this process completely, as previously demonstrated by our group when using ^44^Sc (E_β+av_ = 632 keV, I_β+_ = 94 %, E_γ_ = 1157 keV, I_γ_ = 99.9 %; *T*_1/2_ = 3.97 h) for labeling of the same peptide [23]. It will be of interest to investigate the extent of radiolysis with ^152^Tb-labeled peptides in more detail in future experiments. In addition, comparison with peptides, labeled with conventional PET nuclides like ^68^Ga, would be interesting and allow the development of suitable labeling and storage conditions under the application of commonly used scavengers.

The high sensitivity G8 PET/CT scanner allowed the performance of PET scans with low activities within a short acquisition time. It was, therefore, possible for the first time to perform in vivo PET/CT scans up to 22 h after administration of the ^152^Tb-labeled compound.

The mouse which received 47 MBq ^152^Tb-DOTANOC was also successfully scanned at 32 h after injection as the longest time point of investigation (Additional file 1: Fig. S1).

The PET/CT experiments performed in this study corroborated the hypothesis that ^152^Tb can be used as a diagnostic match to ^177^Lu, as the images reflected what was expected based on biodistribution experiments using ^177^Lu-DOTANOC. It was also demonstrated that the molar amount of injected peptide was crucial with regard to the tumor uptake of radioactivity and, as a result, the tumor-to-kidney ratios. These findings are in agreement with previous findings reported in the literature, which showed that therapeutic quantities of injected peptide may saturate the receptors in somatostatin receptor-positive tissue [24]. In the present study, low peptide amounts (0.5 nmol) showed a clearly higher tumor uptake (~25 % IA/g, 5 h p.i.) as compared to 10-fold higher peptide amounts which resulted in a markedly reduced accumulation in the tumor (~4 % IA/g, 5 h p.i.). These findings were also reflected by the fact that excretion of radioactivity injected into mice was slower if small amounts of peptide were used because, in this case, a larger fraction was accumulated and retained in the tumor tissue (Additional file 1: Fig. S2). Such results are, however, dependent on the tumor type, the tumor size, and its receptor expression level. It may be different for other targeting agents and, hence, such effects have to be carefully evaluated for each single targeting concept and tumor mouse model. In the case of using DOTANOC in AR42J tumor-bearing mice, our results clearly demonstrated how crucial it would be to inject the same peptide amount for diagnosis and therapy in order to allow visualization of the effective tissue distribution expected for the therapeutic match.

While the limited availability of ^152^Tb was considered as a hindrance for further investigations to date, new endeavors are focused on overcoming this obstacle. The present study demonstrated an increase in obtained radioactivity by a factor 30 with respect to our previously published pilot study [1]. In the near future, it is foreseen to increase the ^152^Tb activities further by using thicker tantalum targets that may result in a gain of a factor 2-4, as well as longer collection times resulting in an additional gain of activity of up to a factor 6.

MEDICIS, a new radionuclide production center dedicated to medical applications, is being built at CERN (Geneva, Switzerland). Other facilities exploiting spallation production combined with isotope separation online are under operation at ISAC, TRIUMF (Vancouver, Canada) and IRIS, PNPI (Gatchina, Russia). New facilities are planned or under construction at RIBF (East Lansing, USA), ISOL@MYRRHA (Mol, Belgium), and J-PARC ISOL (Tokai, Japan). Such facilities will provide about two orders of magnitude higher proton current on target compared to the proton current used for the present study, thus, the production of ^152^Tb by spallation and coupled to mass separation has the potential to produce in the range of TBq per day. Such activity would correspond to several hundreds of patient doses. Alternative production at compact cyclotrons via the ^152^Gd (p,n)^152^Tb reaction with 12 MeV protons, followed by radiochemical Gd/Tb separation [25] is feasible once highly enriched ^152^Gd becomes commercially available. One can assume that the more regular production of ^152^Tb will be possible in the near future and provide quantities sufficient for pilot investigations in patients.

## Conclusions

In this study, ^152^Tb was produced at significantly higher quantities (30 times more) than previously achieved. These circumstances allowed the performance of more detailed imaging studies with tumor-bearing mice using in vivo PET/CT. Based on the resulting data, ^152^Tb is a promising candidate to be used as diagnostic match to therapeutic radiolanthanides, potentially allowing exact pre-therapeutic imaging and dosimetry calculations. More regular production of ^152^Tb at high quantities will be crucial to allow further preclinical investigations, as well as pilot studies in the clinics.

## Compliance with ethical standards

### Ethical approval

All applicable international, national, and/or institutional guidelines for the care and use of animals were followed.

This article does not contain any studies with human participants performed by any of the authors.

## Additional file

Additional file 1: (DOC 367 kb)
