# Supplementary material for: Preclinical in vivo application of 152Tb-DOTANOC: a radiolanthanide for PET imaging
Source: EJNMMI Res. 2016 Apr 23;6:35. doi: 10.1186/s13550-016-0189-4 (PMC4842197; doi:10.1186/s13550-016-0189-4)
Supplement: Additional file 1: — (DOC 367 kb) [file 13550_2016_189_MOESM1_ESM.doc]

Supplementary Material

**Preclinical in vivo application of ^152^Tb-DOTANOC: a radiolanthanide for PET imaging**

Cristina Müller^1*^, Christiaan Vermeulen^1^, Karl Johnston^2^, Ulli Köster^3^, Raffaella Schmid^1^, Andreas Türler^4,5^, Nicholas P. van der Meulen^1,4*^

*^1^ Center for Radiopharmaceutical Sciences ETH-PSI-USZ, Paul Scherrer Institute, Villigen-PSI, Switzerland*

*^2^ISOLDE/CERN, Meyrin, Switzerland*

*^3^Institut Laue-Langevin, Grenoble, France*

*^4^Laboratory of Radiochemistry, Paul Scherrer Institute, Villigen-PSI, Switzerland*

*^5^Department of Chemistry and Biochemistry University of Bern, Bern, Switzerland*

* Corresponding authors:

PD Dr. Cristina Müller, Dr. Nicholas P. van der Meulen

Center for Radiopharmaceutical Sciences ETH/PSI/USZ

Laboratory for Radiochemistry

Paul Scherrer Institute

5232 Villigen-PSI

Switzerland

e-mail: [cristina.mueller@psi.ch](mailto:cristina.mueller@psi.ch) and nick.vandermeulen@psi.ch

phone: +41-56-310 44 54 and +41-56-310 50 87

fax: +41-56-310 28 49

**1. Biodistribution study with ^177^Lu-DOTANOC at different peptide amounts**

Biodistribution studies were performed using ^177^Lu-DOTANOC at different molar amounts of peptide in order to investigate the influence on the tissue distribution (Table S1). It was shown that a low peptide amount resulted in a significantly higher tumor uptake (25.3 ± 4.14% IA/g, 5 h p.i.) than a large peptide amount (3.82 ± 0.20 % IA/g, 5 h p.i.), presumably due to receptor saturation effects. In the kidneys the retention of radioactivity was in the same range (~6-9% IA/g) at 5 h after injection of different molar amounts of peptide.

**Table S1 Biodistribution of ^177^Lu-DOTANOC (5 MBq), 5 h after injection of different peptide amounts (0.5 nmol, 2.5 nmol and 5.0 nmol) in AR42J tumor-bearing female nude mice, expressed in percentage of total injected activity per gram tissue (% IA/g)**

|  | ^177^Lu-DOTANOC | | |
| --- | --- | --- | --- |
|  | 0.5 nmol | 2.5 nmol | 5 nmol |
| Blood | 0.09 ± 0.01 | 0.06 ± 0.01 | 0.06 ± 0.01 |
| Heart | 0.08 ± 0.01 | 0.07 ± 0.01 | 0.06 ± 0.00 |
| Lung | 1.18 ± 0.36 | 0.41 ± 0.04 | 0.28 ± 0.01 |
| Spleen | 0.24 ± 0.03 | 0.18 ± 0.02 | 0.11 ± 0.02 |
| Kidneys | 6.63 ± 0.10 | 9.10 ± 1.66 | 7.00 ± 0.69 |
| Adrenals | 0.70 ± 0.13 | 0.37 ± 0.05 | 0.18 ± 0.05 |
| Stomach | 3.78 ± 0.28 | 1.21 ± 0.55 | 0.41 ± 0.05 |
| Pancreas | 2.70 ± 0.21 | 0.88 ± 0.11 | 0.41 ± 0.01 |
| Intestines | 0.54 ± 0.08 | 0.25 ± 0.03 | 0.14 ± 0.01 |
| Liver | 0.34 ± 0.01 | 0.53 ± 0.10 | 0.36 ± 0.04 |
| Muscle | 0.02 ± 0.01 | 0.02 ± 0.01 | 0.02 ± 0.00 |
| Bone | 0.33 ± 0.08 | 0.12 ± 0.02 | 0.09 ± 0.02 |
| Brain | 0.01 ± 0.00 | 0.02 ± 0.00 | 0.01 ± 0.00 |
| AR42J Tumor | 25.3 ± 4.14 | 8.45 ± 1.04 | 3.82 ± 0.20 |
| Tumor-to-blood | 265 ± 26.5 | 137 ± 15.3 | 62.4 ± 5.81 |
| Tumor-to-liver | 73.6 ± 11.9 | 16.2 ± 2.10 | 10.7 ± 1.13 |
| Tumor-to-kidney | 3.82 ± 0.67 | 0.94 ± 0.16 | 0.55 ± 0.05 |

values shown represent the mean ± S.D. of data from three animals (n=3) per cohort

**2. PET/CT scans 32 h after injection of ^152^Tb-DOTANOC**

A PET/CT scan of an AR42J tumor-bearing mouse was performed 32 h after injection of ^152^Tb-DOTANOC (47 MBq, 4.7 nmol) (Fig. S1). Determination of the radioactivity in the mouse at scan start revealed ~0.45 MBq. The tumor-to-kidney ratio of accumulated radioactivity was determined using *VivoQuant* software resulting in a value of ~1.1 which was equal to what was determined for the PET image taken 22 h after injection of ^152^Tb-DOTANOC (see main manuscript Fig. 6A).

**
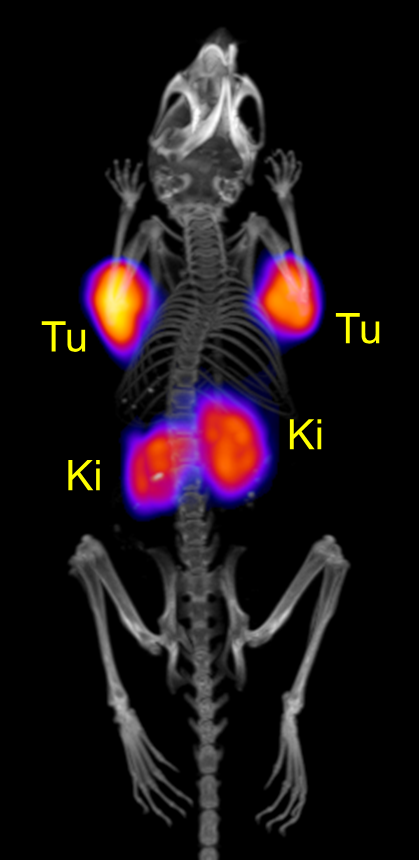
**

**Fig. S1:** PET/CT image shown as maximal intensity projections of an AR42J tumor-bearing mouse 32 h after injection of ^152^Tb-DOTANOC (47 MBq; 4.7 nmol DOTANOC). The PET scan lasted for 30 min and was followed by a CT scan of 1.5 min. During the in vivo scan the mouse was anesthetized with a mixture of Isoflurane and oxygen. (Tu = AR42J tumor xenograft, Ki = kidney)

**3. In vivo clearance of ^177^Lu-DOTANOC**

AR42J tumor-bearing mice were injected with ^177^Lu-DOTANOC at molar amounts of 0.5 nmol (n = 2), 2.5 nmol (n = 2) and 5.0 nmol (n = 2). Each mouse was measured immediately after injection of the radiopeptide and the determined value was set to 100%. Afterwards the mice were measured at different time points after injection which allowed determination of the percentage of injected activity (% IA) retained in the body. Combination of the data points and decay correction revealed excretion curves for each group (Fig. S2). The excretion of radioactivity was faster if large peptide amounts were injected than if only small peptide amounts were employed. Based on the tissue distribution studies, the tumor uptake was significantly increased if only small amounts of peptide were used. As a logical consequence a high tumor uptake (and retention) resulted in a slower wash-out of radioactivity from the whole body. The value may, however, vary from mouse to mouse as it is dependent on the tumor size.


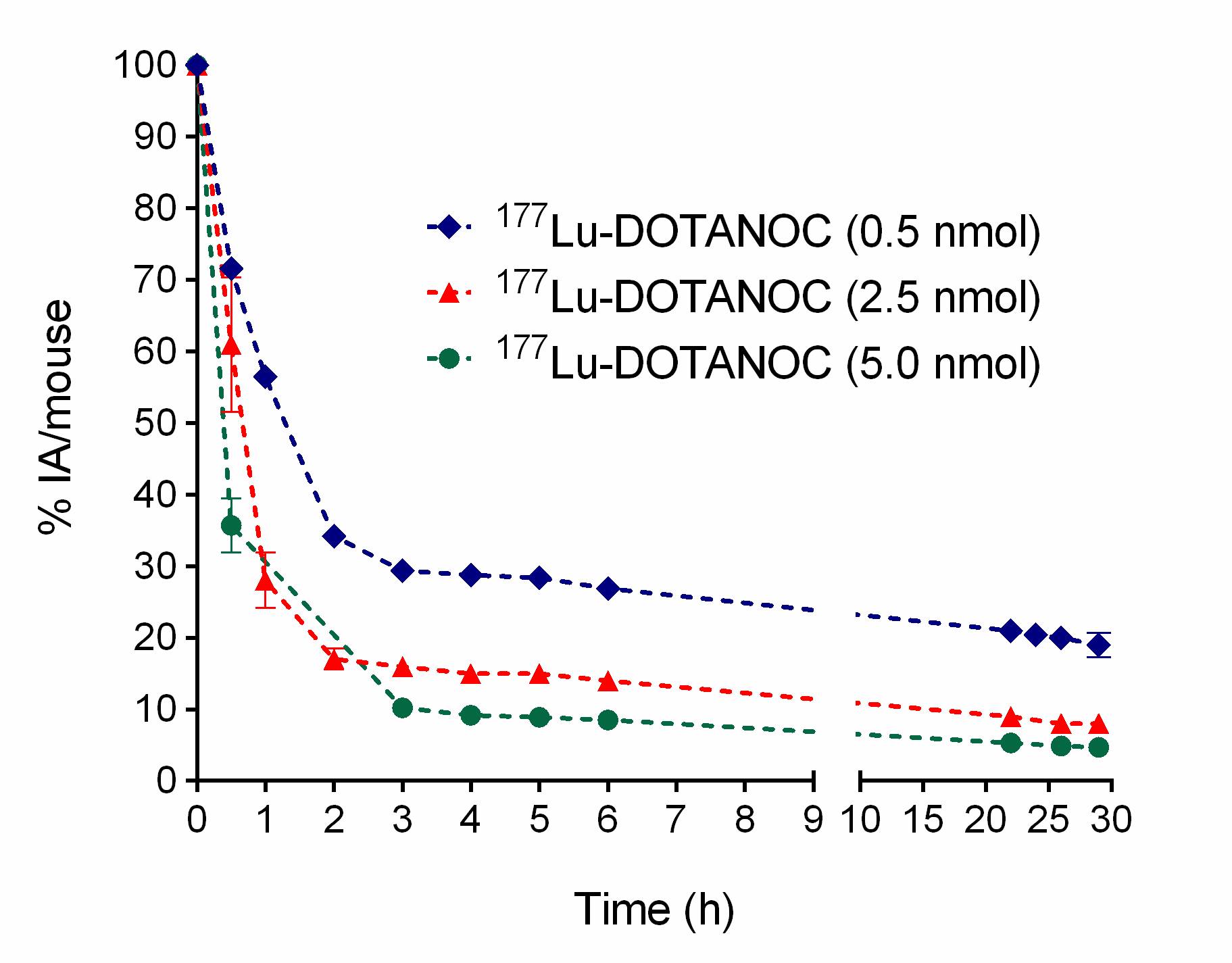


**Fig. S2** Retention of radioactivity in AR42J tumor-bearing mice over time after injection of ^177^Lu-DOTANOC using variable amounts of peptide (0.5 nmol, 2.5 nmol and 5.0 nmol).
